# A description of the current status of chronic fatigue syndrome and associated factors among university students in Wuhan, China

**DOI:** 10.3389/fpsyt.2022.1047014

**Published:** 2023-01-12

**Authors:** Lunbing Luo, Yutong Zhang, Tao Huang, Fang Zhou, Change Xiong, Yang Liu, Piyong Zhai, Guiping Wang, Jianhua Tan, Chengjun Jiao, Xin Chen, Jiao Yu, Yuhao Qiao, Shuqi Ren, Xiaohui Hu, Jianbo Zhan, Jing Cheng

**Affiliations:** ^1^School of Public Health, Wuhan University of Science and Technology, Wuhan, Hubei, China; ^2^The Third People’s Hospital of Hubei Province, Wuhan, Hubei, China; ^3^Chinese Center for Disease Control and Prevention, Beijing, China; ^4^Huanggang City Center for Disease Control and Prevention, Huanggang, China; ^5^Hubei Provincial Center for Disease Control and Prevention, Wuhan, Hubei, China; ^6^Hubei Key Laboratory of Occupational Hazard Identification and Control, Wuhan University of Science and Technology, Wuhan, Hubei, China

**Keywords:** chronic fatigue syndrome, university students, associated factors, anxiety, depression

## Abstract

**Introduction:**

Myalgic encephalomyelitis/chronic fatigue syndrome (ME/CFS) is a group of chronic conscious fatigue that is not easily relieved by rest and is accompanied by corresponding physiological dysfunction and psychological symptoms. However, due to the insufficient understanding of the pathogenesis of ME/CFS, there is currently a lack of effective treatment methods. In addition, there are few surveys on the current status of ME/CFS in the central region of China, and the data on ME/CFS among university students in the central region are lacking. This group conducted a survey on university students in Wuhan, Hubei Province in 2022 to collect and analyze the current status of ME/CFS among university students in central China for the first time, aiming to understand the current development of ME/CFS among university students, investigate the influencing factors of its prevalence, fill the data gaps, and provide a reliable basis for developing interventions for chronic fatigue syndrome among university students.

**Methods:**

A cross-sectional study was conducted among university students in a university in Hubei province. Data were collected via online questionnaire surveys. The contents included demographic characteristics, lifestyles, disease history, depression, anxiety, sleep, ME/CFS and other associated factors. SAS 9.4 statistical software was used to analyze and estimate the effect of associated factors on ME/CFS.

**Results:**

A total of 1826 subjects were included in the final analysis. The results showed that the prevalence of ME/CFS in university students was 6.25%. Univariate analysis showed that exercise, alcohol consumption, study, overnights, diet, anxiety, depression, and sleep quality were associated with ME/CFS (*P* < 0.05). Multivariate analysis showed that overnights, overeating, anxiety, and sleep quality were independent risk factors, while learning was a protective factor.

**Conclusion:**

College students should pay enough attention to ME/CFS, improve their understanding of ME/CFS, and improve people’s ability to understand ME/CFS.

## 1. Introduction

Myalgic encephalomyelitis/chronic fatigue syndrome (ME/CFS) is a group of chronic conscious fatigue that is not easily relieved by rest and is accompanied by corresponding physiological dysfunction and psychological symptoms (1). Since 1988, when the Centers for Disease Control and Prevention (CDC) formally proposed the diagnostic criteria for ME/CFS, people have gradually found that this disease widely exists in the whole population (2).

At present, the prevalence of chronic fatigue syndrome in the global general population is between 0.1 and 2.2% (3). The survey of Lim et al. (2) retrieved ME/CFS prevalence data from 1980 to December 2018, the results showed that the prevalence of ME/CFS in the American population is between 0.89 and 1.14%. A cohort study in the United Kingdom in 2017 showed that the prevalence of ME/CFS in adolescents ranged from 1.47 to 2.99% ME/CFS (4). A recent meta-analysis in China showed that the prevalence of ME/CFS in China was 12.54% (5). Among patient with ME/CFS, there are more female patients than male patients, the proportion of young and middle-aged patients is higher than that of other age groups, and the proportion of people with high education is higher than that of people with low education (6).

Several studies have shown that the symptoms of cognitive impairment in patient with ME/CFS are universal and typical. Clinically, many patients with CFS have symptoms of cognitive function changes, especially memory decline and inattention, accompanied by gastro intestinal disorders and food intolerance (7, 8). However, the heterogeneity and concealment of CFS delay the diagnosis and treatment of patients with this disease to varying degrees. According to a report (9) published by the American Institute of Medicine (IOM) in 2015, it is estimated that there are 83,600–2.5 million people suffering from CFS in the United States, most of whom have not been clinically recognized, and at least a quarter of whom are sick in bed because they have not received effective treatment. Therefore, the invisible status of ME/CFS is not only an individual health problem but also a serious public health problem (10).

Studies (11) have shown that ME/CFS, as an independent disease, is different from depression, anxiety, and other mental diseases, but it is closely related to depression, anxiety, and other emotional disorders. The fatigue degree of patient with ME/CFS is directly proportional to the scores of depression and anxiety, and the two can influence each other to promote the progression of the disease. According to the statistics, at least one-fifth of adolescents with chronic fatigue syndrome also have depression, and one-fourth of adolescents with ME/CFS also meet the criteria of at least one anxiety disorder (12), and emotional disorders, such as depression and anxiety, can increase the suicide risk of patient with ME/CFS to a certain extent (13, 14).

According to a recent meta-analysis (5), the prevalence of ME/CFS among university students is 13.38%, which is the highest prevalence among the different education groups. Xing Hanwei et al. conducted a survey on university students in Zhejiang Province in 2018, which showed that 20.2% of university students were detected with ME/CFS (15), an increase compared with the 5.4% prevalence rate in a university in Guangxi in 2011 (16). At present, most university students are faced with high pressure and a fast pace of studying and life, with relatively rich mental activities and less daily health exercise, which makes ME/CFS a disease that is easy to occur among university students and affects their life and study.

The pathogenesis of ME/CFS are still unclear in modern medicine, and it is speculated that the etiology may be related to infection, immune abnormalities, endocrine and metabolic disorders, and nervous system inflammation (17–19). However, due to the insufficient understanding of the pathogenesis of ME/CFS, there is currently a lack of effective treatment methods. In addition, there are few surveys on the current status of ME/CFS in the central region of China, and the data on ME/CFS among university students in the central region are lacking.

This group conducted a survey on university students in Wuhan, Hubei Province in 2022 to collect and analyze the current status of ME/CFS among university students in central China for the first time, aiming to understand the current development of ME/CFS among university students, investigate the influencing factors of its prevalence, fill the data gaps, and provide a reliable basis for developing interventions for chronic fatigue syndrome among university students.

## 2. Materials and methods

### 2.1. Research subjects

The subjects of this investigation had the right to informed consent and signed informed consent forms before being investigated, and participated voluntarily. The research subjects were all university students studying at Wuhan University of Science and Technology from May 2022 to July 2022.

The inclusion criteria were as follows: (1) Patients have good cognitive status, no verbal communication impairment, and can fully cooperate with the research process; (2) Did not receive any treatment for fatigue, including taking health supplements, within one month before the study; (3) Volunteer to participate in this study and sign the informed consent form.

The exclusion criteria were as follows: (1) Have a known medical condition such as epilepsy, inflammatory bowel disease, diabetes mellitus, chronic obstructive pulmonary disease, or rheumatoid arthritis; (2) Have other causes of fatigue such as post-cancer fatigue and other conditions that may cause severe fatigue. (3) Students who are unwilling to cooperate with the investigation or are participating in another study, which may affect the results of this study.

### 2.2. Investigation methods

This study used the stratified cluster random sampling method to conduct an online questionnaire survey among university students at Wuhan University of Science and Technology. Taking grade as a stratification factor, 10 classes of each grade in Wuhan University of Science and Technology were randomly selected, and all the students in the classes were investigated using questionnaires. A total of 1,826 questionnaires were distributed and 1,826 were recovered (a recovery rate of 100%). After removing 34 invalid questionnaires, 1,792 valid questionnaires were obtained, and the effective rate was 98.14%.

The study was conducted in accordance with the Helsinki declaration and approved by the Ethics Committee for Medical Research, School of Public Health, Wuhan University of Science and Technology; the ethics approval number is 2022125. Written informed consent was obtained from all participants or their legal guardians before the investigation. The questionnaire used in our study was developed for this study.

### 2.3. Questionnaire

According to the diagnostic criteria for ME/CFS established by the CDC of America (20), the self-designed “University Students Chronic Fatigue Syndrome Questionnaire”^1^ was used to investigate the general situation, factors associated with ME/CFS, fatigue, depression, anxiety, and sleep quality of students in Wuhan University of Science and Technology. The general information included gender, age, grade, major, etc. The associated factors of ME/CFS included three aspects: lifestyle, learning, and social support.

Fatigue Scale (21) (FS-14): The closed and two-point question forms were used to calculate the scores. They included 14 items in total. The scores of the first to the eighth items were summed to obtain the physical fatigue score, with the highest score of 8. The scores of the 9th to the 14th items were summed to obtain a mental fatigue score, with a maximum of 6 points. The total fatigue score was calculated by adding the physical and mental fatigue scores, with a maximum of 14. A higher score implied a higher degree of fatigue.

The Sleep Quality Scale (22) (PSQI) was developed in 1989 by Dr. Buysse et al., a psychiatrist at the University of Pittsburgh. The sensitivity and specificity of the PSQI were 98.3 and 90.2%, respectively (Kappa = 0.89, *P* < 0.01) (23). This scale is suitable for the evaluation of the sleep quality of patients with sleep and mental disorders, and also for the evaluation of the sleep quality of the general population. It was used to assess the sleep quality of the subjects in the last month. The higher the score, the worse the sleep quality.

The psychological status was assessed using the Self-rating Depression Scale (24) (SDS) and the Self-rating Anxiety scale (25) (SAS). The SDS and SAS scales are commonly used in psychopharmacology research. There are 20 questions in total, and subjects are required to make an independent assessment, which takes about 10 min to complete. The scores of each question are added to the rough score, which is multiplied by 1.25 and rounded up to a standard score. The higher the score, the more severe the anxiety or depression. The upper limit reference value of the SDS standard score is 53; <53 is normal, 53–62 is mild depression, 63–72 is moderate depression, and 72 and above is severe depression. The upper limit reference value of the SAS standard score is 50; <50 is normal, 50–60 is mild anxiety, 61–70 is moderate anxiety, and >70 is severe anxiety.

### 2.4. Diagnostic criteria

This was according to the Fukuda standard revised by the Centers for Disease Control and Prevention (CDC) in 1994 (20), which includes the following two aspects:

(1) Medically unexplained recurrent or persistent episodes of debilitating fatigue, of at least six months duration, for unknown reasons, that basically cannot ease after resting, is of new onset (not life-long from childhood), and leads to an obvious decline in a person’s usual activity level (e.g., social or personal life, work, study).
(2) There are nine entries for ME/CFS symptoms; a patient with ME/CFS has four or more of the following at the same time and these symptoms persist or occur frequently for six months or more: ➀ prolonged fatigue for which no cause can be found and which cannot be relieved after rest; ➁ inability to recover energy after sleep; ➂ swelling and tenderness of lymph nodes in the neck or axilla; ➃ muscle pain and stiffness; ➄ pain in multiple joints without swelling or redness; ➅ new or sudden onset of headache; ➆ frequent or recurrent sore throat; ➇ poor memory and concentration or significant loss of concentration; ➈ physical discomfort 24 hours after physical or mental work.

### 2.5. Statistical analysis

SAS 9.4 statistical analysis software was applied for data analysis. Descriptive statistics were performed for basic demographic characteristics, fatigue scores, anxiety, depression status, and sleep quality. Fatigue scores, anxiety, depression, and sleep quality were continuous variables, expressed as means ($\bar{\text{x}}$ ± s), and comparisons between groups were analyzed using ANOVA or non-parametric tests if they did not meet normality. Binomial logistic regression was used to analyze the relationship between ME/CFS and other factors, and logistic regression equations were constructed to determine the predictive effect of each factor.

The test level α for this study was set at 0.05, and *P* < 0.05 was considered statistically significant.

## 3. Results

### 3.1. Analysis of the prevalence and basic conditions of ME/CFS

A questionnaire survey was conducted among 1,792 university students, among whom 112 patients with ME/CFS were detected, and the prevalence rate was 6.25%. The chi-square test was used to compare the prevalence of ME/CFS among respondents of different ages, genders, and educational backgrounds. The results showed that there was no significant difference in the prevalence of ME/CFS among different genders, ages, grades, and majors (*P* > 0.05; Table 1).

**TABLE 1:** Prevalence and characteristics of ME/CFS.

Variable	*n*	CFS	Non-CFS	Prevalence (%)	*X* ^2^	*P*
**Gender**						
Male	992	67	925	6.75	0.96	0.33
Female	800	45	755	5.63		
**Age**						
<19	326	30	296	9.20	6.02	0.11
19–22	1,198	66	1,132	5.51		
22–25	164	10	154	6.10		
26–29	104	6	98	5.77		
**Grade**						
Fresh year	673	52	621	7.73	4.42	0.35
Sophomore year	626	33	593	5.27		
Junior year	139	7	132	5.04		
Senior year	137	9	128	6.57		
Others	217	11	206	5.07		
**Major**						
Medical	715	41	674	5.73	3.8991	0.14
Science and engineering	875	52	823	5.94		
Others	202	19	183	9.41		

### 3.2. Fatigue Scale (FS-14) scores and univariate analysis

We analyzed the differences in the Fatigue Scale (FS-14) scores of the university students in basic demographic characteristics, lifestyle, learning, and social support. Wilcoxon rank sum test and Kruskal-Wallis test were used for comparison (if data did not conform to normality). The results showed that the difference in mental fatigue between sexes was statistically significant (*P* < 0.05), and men were more likely to have mental fatigue than women. In terms of lifestyle, the effects of exercise, drinking, diet, and sleep on fatigue were statistically significant (*P* < 0.05). The scores of fatigue were lower in people who had regular exercise, paid attention to sugar control, maintained weight, and did not stay up all night. Alcohol consumption increased fatigue scores. Eating regularly, not overeating, eating fewer snacks, drinking less milk tea, eating meat in combination with vegetables, and eating fewer pickles reduced the fatigue score. The longer the study time, the lower the fatigue score was. In terms of social support, when encountering difficulties, often seeking help, participating in group activities, and being able to express emotions freely reduced the fatigue score. Subjects with high fatigue scores often had headache, dizziness, nausea, dyspnea, numbness and tingling, and heavy hands and feet (Supplementary Table 1).

### 3.3. Effects of anxiety and depression on fatigue

The effects of depression and anxiety on fatigue were analyzed using a factorial design. The results showed that there was a statistically significant difference in fatigue scores between the anxiety group and the no-anxiety group (*F* = 68.83, *P* < 0.05), and a statistically significant difference in fatigue scores between the depression group and the no-depression group (*F* = 260.42, *P* < 0.05), while the interaction between depression and anxiety was not statistically significant (*F* = 1.0, *P* = 0.32). This indicates that the effects of anxiety and depression on fatigue are independent, that is, a change in the level of one factor does not affect the effect of the other factor on fatigue (Table 2).

**TABLE 2:** Effects of depression and anxiety on fatigue score (FS-14).

			**SDS**		***F***	***P***
			**Depression**	**Non-depression**		
SAS	Anxiety	*n*	1,041	76	68.83	<0.0001
		−x ± s	3.9 ± 2.9	5.9 ± 3.0		
	Non-anxiety	n	310	365		
		−x ± s	5.6 ± 3.5	7.2 ± 3.6		
*F*			260.42			
*P*			<0.0001			

The correlations between anxiety, depression, and fatigue scores were studied. The results showed that anxiety and depression were significantly correlated with fatigue. The correlation coefficient between anxiety and fatigue was *r* = 0.94, and the correlation coefficient between depression and fatigue was *r* = 0.94; both were statistically significant (*P* < 0.05). After adjusting for the effects of depression and anxiety on the correlation, the correlation coefficients were both 0.98, which were still statistically significant (*P* < 0.05; Table 3).

**TABLE 3:** Spearman correlation analysis of depression, anxiety, and fatigue score (FS-14).

	SDS	SAS	FS-14
SDS	1.00	0.77**	0.94**
SAS		1.00	0.94**
FS-14			1.00

**P < 0.0001.

### 3.4. Multivariate analysis of ME/CFS

ME/CFS is affected by a variety of related factors, and the analysis of a single factor cannot control the influence of confounding factors. Therefore, multivariate logistic regression analysis was used to explore the influencing factors of ME/CFS in university students. With ME/CFS detection or not as the dependent variable and eight factors (including exercise, alcohol consumption, study, overnights, dietary patterns, anxiety, depression, and sleep quality) as independent variables, the relevant variables were assigned (Supplementary Table 2). After multi-factor logistic regression analysis, the results showed that study, overnights, diet, anxiety, and sleep quality were associated factors for ME/CFS, with overnights, overeating, anxiety, and sleep quality as independent risk factors and study as a protective factor. The differences in the prevalence of ME/CFS by exercise and alcohol consumption were not statistically significant (Table 4).

**TABLE 4:** Multi-factor logistic regression analysis of CFS.

	Variables	β	S. E.	Wald χ^2^	*P*	OR	95% CI	
Study	1–2 h	−0.624	0.30	4.47	0.035	0.535	0.300	0.956
	2–4 h	−1.091	0.35	9.74	0.002	0.336	0.169	0.666
	>4 h	−0.176	0.29	0.40	0.527	0.839	0.486	1.447
Overnight	Sometimes	0.061	0.23	0.07	0.787	1.063	0.683	1.654
	Often	0.881	0.33	7.34	0.007	2.414	1.276	4.567
Overeating		0.409	0.20	4.09	0.043	1.504	1.013	2.235
SAS		0.702	0.21	11.32	0.001	2.017	1.340	3.036
PSQI		1.671	0.22	59.89	<0.001	5.318	3.482	8.122

## 4. Discussion

In recent years, more and more scholars have begun to study chronic fatigue syndrome. The prevalence of ME/CFS varies in different countries. The prevalence of ME/CFS in the United States ranged from 0.89 to 1.14% (2). ME/CFS. The prevalence rates of ME/CFS in Japan and South Korea are 0.76 and 0.77%, respectively (26). The meta-analysis of Wu Qiaoqiao et al. (5) showed that the total prevalence of ME/CFS in the Chinese population is 12.54%, which was significantly higher than those in other countries. The possible reason is that most of the literature included in the meta-analysis by Wu Qiaoqiao et al. are of studies on high-risk groups such as teachers, nurses, and students, which leads to the high prevalence of ME/CFS in China, and there is still a lack of studies on the prevalence of ME/CFS in the general population in China. This study investigated the prevalence of ME/CFS among university students at Wuhan University of Science and Technology. The prevalence rate was 6.25%, which was lower than the national average level. In China, university students have been reported to have the highest prevalence of ME/CFS among all academic groups. The prevalence of ME/CFS in a university in Zhejiang province was as high as 20.2% (15), and the prevalence of ME/CFS in medical students in Jiangxi and Hunan provinces reached 17.55% (27). Most scholars believe that medical students have a higher prevalence of ME/CFS, but in this study, there was no statistically significant difference in the prevalence of ME/CFS between medical students and students of other majors.

When it comes to gender, reports vary from country to country. In a study in Korea, the prevalence rate of ME/CFS among women was twice that among men, while in a study in Japan, the gender difference between men and women was not obvious (26). A national cohort study in Spain showed that women were at high risk for ME/CFS (28). According to a British study, post-adolescent girls have a higher risk of ME/CFS (29). In this study, there was no statistically significant difference in the prevalence of ME/CFS by gender. However, there was a statistically significant difference in the mental fatigue score between men and women in the Fatigue scale (FS-14), and the mental fatigue score of women was greater than that of men. The fatigue difference between men and women may be influenced by the physiological and psychological characteristics of women. First of all, women are prone to gynecological diseases, and many studies have shown that ovarian cysts, uterine fibroids, menstrual abnormalities, endometriosis, etc., are risk factors for ME/CFS (30, 31). Second, female university students are at a higher level of the female group. Compared with male students, they are faced with a more complex environment and opportunities. On the one hand, they are bound by the traditional national culture and the environmental atmosphere created, bearing the heavy burden of male superiority. On the other hand, the requirements of modern society and the development of a market economy make them and boys alike enter the competition and struggle for living space. In addition, their inner conflicts, contradictions, and imbalances may be more complex, intense, and hidden than those of boys.

This study found that university students’ lifestyle behaviors, such as exercise, drinking, and staying up all night, could affect the prevalence of ME/CFS. People who exercised regularly, took naps, and did not stay up all night had lower fatigue scores, according to the Fatigue Scale (FS-14). Exercise can not only reduce physical fatigue but also reduce mental fatigue. Xu (32) selected 70 patients with ME/CFS as experimental subjects through clinical experiments. The exercise group carried out progressive exercise, while the control group did not exercise. The results showed that proper and regular exercise can achieve the ideal effect of treating chronic fatigue syndrome.

Siestas and getting enough sleep at night can also reduce feelings of fatigue. The more participants stayed up all night, the more tired they felt, and those who took naps had lower fatigue scores than those who did not. This conclusion is consistent with the research of Wei et al. (33). Some scholars believe that the occurrence of ME/CFS is closely related to the change in sleep rhythm (34). Most clinical manifestations of patient with ME/CFS are similar to the symptoms of rhythm disorder, and the change in rhythm leads to sleep disorders, fatigue, cognitive impairment, etc. (35). By comparing the circadian rhythm patterns of rest and activity between patient with ME/CFS and healthy individuals, foreign scholars found that the stability of daily activities and activity rhythm of patient with ME/CFS was lower than that of the control group, and concluded that the symptoms of ME/CFS could be effectively alleviated by regulating the circadian rhythm (36).

Although fatigue is often the main clinical manifestation of ME/CFS, it is often severe insomnia that compels patients to seek medical attention. Sleep disorders not only delay the daily work of patients but also have a negative impact on their lives. In the longterm, they will make patients feel anxious and progress to depression in serious cases. Several studies have shown that sleep disorders have a certain correlation with anxiety and depression (19, 37, 38). Atthe same time, this study found that fatigue degree is also strongly correlated with depression and anxiety (*r* = 0.98, *P* < 0.05). Although anxiety and depression were correlated (*r* = 0.77, *P* < 0.05), the factorial analysis showed that anxiety and depression had independent effects on fatigue. Therefore, improving sleep disorders, relieving anxiety and depression, and reducing fatigue are of great significance in the treatment of ME/CFS.

In terms of eating habits, a regular diet can reduce the likelihood of ME/CFS. People with “no specific meal times” are more likely to be fatigued than those with “regular meal times”; those who prefer overeating, snacking, pickles, and drinking milk tea have higher fatigue levels; while those who usually pay attention to the nutrition table of food, control sugar intake, control weight, and eat meat in combination with vegetables have lower fatigue levels. ME/CFS is mostly accompanied by the irrational diet content, incorrect understanding of health concepts, and the existence of unhealthy lifestyle habits. Although neither Campagnolo (39) nor Jones et al. (40) in their review of observational studies found sufficient evidence that dietary intake patterns and vitamins or minerals alleviate ME/CFS, Jones suggested that chronic fatigue syndrome is characterized by increased indoor residence, potentially increased risk of osteoporosis, and significantly lower mean serum 25-OH vitamin D levels. Regardless of the results of the study, vitamin D intake through diet and supplements should be considered for general health.

Social support mainly refers to the psychological and material conditions and resources that individuals can obtain through interpersonal networks (41). Many studies have concluded that social support is also an important factor influencing the prevalence of ME/CFS. In the current study, it was found that people who frequently seek help when they encounter troubles, regularly participate in group activities, and are able to express their emotions comfortably tend to have lower fatigue rates. It has been suggested that social support affects not only mental health but also physical health. Social support is especially important at work because it may make people experience work more positively, while those who approach their work with negative emotions tend to suffer from mental or physical illnesses (42). Nowadays, online communities have become an important communication channel for people with illnesses or health risk groups to seek social support. Sun et al. (41) found that in online communities focusing on psychological and mental health problems, exchanging and sharing emotions, experiences, and stories with other members, rather than simply asking for help with direct information, was the most powerful social support for patients. One study indicated that online social activities through online health communities were beneficial for improving or maintaining health behaviors and had positive effects on behaviors such as diet and exercise, while the study categorized social relationships into friendship, mutual support, and competition and found that friendship had the greatest effect on health behaviors (43).

In this study, the factors influencing ME/CFS were analyzed by binary logistic regression, and it was found that study was a protective factor for ME/CFS, and the OR was smallest when the study time of university students was 2–4 h (OR = 0.34, *P* < 0.05), but the OR increased and the protective effect weakened as the study time increased. This phenomenon may be due to the fact that fatigue caused by prolonged learning reduces the protective effect of learning, and maintaining an appropriate and reasonable learning time can effectively reduce the possibility of ME/CFS occurrence. Some scholars have compared the differences between the spatial learning and memory functions of ME/CFS mice and normal mice by replicating the chronic fatigue syndrome (ME/CFS) mouse model through the compound stimulation method, and the results showed that the spatial learning and memory functions of ME/CFS mice were reduced (44). In turn, the reduced learning function leads students to spend more time studying, which in turn induces the development of ME/CFS, creating a vicious cycle.

In addition, due to the epidemic of COVID-19 in recent years, it has caused varying degrees of impact on human life and health. A Japanese study included 279 patients who met the long-COVID found that their overall prevalence of ME/CFS was 16.8%, and most of them had characteristic symptoms of ME/CFS such as dizziness, chest pain, insomnia, and headache (45). Some scholars believe that the long-COVID syndrome, especially brain fog, may be related to nervous system inflammation (46), and nervous system inflammation is one of the causes of CFS (18), indicating that there is a correlation between COVID-19 and CFS in symptoms and causes. If COVID-19 develops for a long time, it may progress to CFS. Therefore, it is very important to effectively prevent and treat CFS and explore the related risk factors of CFS.

## 5. Conclusion

Because ME/CFS has many first symptoms and less specificity, it is easily ignored by patients and physicians clinically, which leads to the progression of the disease. Therefore, it is important to increase the knowledge about ME/CFS, improve the awareness of ME/CFS, and intervene against risk factors to prevent and treat this disease. In this study, by analyzing data on general conditions, lifestyle, eating habits and social support of students at Wuhan University of Science and Technology, overnight, overeating, anxiety and sleep quality were considered as independent risk factors and appropriate study as protective factors, for which the following recommendations were made ME/CFS: (1) ME/CFS in university students needs to be given sufficient attention. It is also rare to investigate and evaluate the prevalence of ME/CFS in the domestic population of university students; (2) we should continue to strengthen the research on ME/CFS, especially the influence of dietary and psychosocial factors on ME/CFS; (3) we should strengthen the health awareness of university students, develop good living habits, and strengthen the interventions on the mental health of university students.

## 6. Strength and limitations

(1) This study only analyzed the current situation and influencing factors of chronic fatigue syndrome among university students at Wuhan University of Science and Technology, and the results cannot be extrapolated to the whole country, but the study had a relatively large sample size to ensure the reliability of the results. (2) This study is a cross-sectional study, and the causal relationship between the influencing factors and ME/CFS cannot be judged, so it is necessary to carry out further intervention studies and evaluate the effect of lifestyle according to the characteristics of the students. (3) The recent epidemic of COVID-19 and CFS showed similar characteristics, namely physical and mental fatigue. However, because none of the subjects selected during the study period were infected with COVID-19, this study could not analyze the effect of COVID-19 on CFS.

## Data availability statement

The original contributions presented in this study are included in the article/Supplementary material, further inquiries can be directed to the corresponding authors.

## Ethics statement

The studies involving human participants were reviewed and approved by Wuhan University of Science and Technology. The patients/participants provided their written informed consent to participate in this study.

## Author contributions

JC conceived and designed the study. LL, YZ, TH, and FZ wrote the manuscript. CX, YL, PZ, GW, JT, and CJ offered us epidemiological investigation strong support. XC, JY, YQ, SR, XH, and JZ involved in the study. All authors contributed to the discussions and wrote the manuscript.

## Footnotes

1
https://www.wjx.cn/vj/YD3tgDF.aspx
