# Supplementary material for: A description of the current status of chronic fatigue syndrome and associated factors among university students in Wuhan, China
Source: Front Psychiatry. 2023 Jan 12;13:1047014. doi: 10.3389/fpsyt.2022.1047014 (PMC9877457; doi:10.3389/fpsyt.2022.1047014)
Supplement: Supplementary file 1 [file Data_Sheet_1.PDF]

Self-rating Anxiety Scale(SAS)

Please read each entry carefully to understand its meaning, and then cross it in the appropriate box according to your actual situation in the last week.

1. I feel more easily stressed or anxious than usual
2. I'm scared for no reason.
3. I get upset or frightened easily
4. I think I might be going crazy.

*5. I think everything is fine

6. my hands and feet tremble and shake
7. I struggle with headaches, neck pain and back pain
8. I feel easily debilitated and tired

*9. I feel calm and easy to sit quietly

10.I feel my heart beating fast.

11. I was distressed by a bout of dizziness

12. I have fainting episodes, or feel like I'm going to faint

*13. I breathe in and out so easily

14.Numbness and tingling in my hands and feet

15. I struggle with stomach pain and indigestion

16. I often have to pee

*17. My hands and feet are often dry and warm

18. I blush and get hot

* 19. I fall asleep easily and sleep well through the night

20. I have nightmares

Scoring: Forward scoring questions A, B, C, and D are scored as 1, 2, 3, and 4; reverse scoring questions are scored as 4, 3, 2, and 1. Reverse scoring question numbers: 5, 9, 13, 17, 19 (those marked with *). The total score is multiplied by 1.25 and rounded up to the nearest whole number to obtain the standard score, the smaller the score, the better.
