# Supplementary material for: A description of the current status of chronic fatigue syndrome and associated factors among university students in Wuhan, China
Source: Front Psychiatry. 2023 Jan 12;13:1047014. doi: 10.3389/fpsyt.2022.1047014 (PMC9877457; doi:10.3389/fpsyt.2022.1047014)
Supplement: Supplementary file 2 [file Data_Sheet_2.PDF]

Self-rating Depression Scale(SDS)

Note: There are 20 items of text below. Please read each item carefully to understand its meaning, and then cross it in the appropriate box according to your last week's actual situation.

1. I feel sullen and depressed.

* 2. I think the morning is the best part of the day.

3.I burst into tears or feel like crying.

4. I have trouble sleeping at night.

*5. I ate as much as I normally do.

* 6. I feel as happy as ever when I am in close contact with the opposite sex.

7. I find that I am losing weight.

8. I suffer from constipation.

9. My heart is beating faster than usual.

10. I feel tired for no reason.

*11. my mind is as clear as usual.

*12. I do not find it difficult to do what I often do.

13. I feel restless and unsettled.

* 14. I have hope for the future.

15. I get angry and agitated more easily than usual.

*16. I find it easy to make decisions.

*17. I feel useful and that I am needed.

*18. I've had an interesting life.

19. I think others would be better off if I were dead.

*20. What normally interests me still interests me as usual.

Scoring: Forward scoring questions A, B, C, and D are scored as 1, 2, 3, and 4; reverse scoring questions are scored as 4, 3, 2, and 1. Reverse scoring question numbers: 2, 5, 6, 11, 12, 14, 16, 17, 18, 20 (marked with *). The standard score is obtained by multiplying the total score by 1.25 to the nearest whole number, the smaller the score, the better.
