# Supplementary material for: A description of the current status of chronic fatigue syndrome and associated factors among university students in Wuhan, China
Source: Front Psychiatry. 2023 Jan 12;13:1047014. doi: 10.3389/fpsyt.2022.1047014 (PMC9877457; doi:10.3389/fpsyt.2022.1047014)
Supplement: Supplementary file 3 [file Data_Sheet_3.pdf]

Questionnaire on Chronic fatigue Syndrome in college students

Dear Students,

Hello!

We are the investigators of the "Chronic Fatigue Syndrome (CFS) Epidemiological Investigation" group, students from the medical school. Today we will join you in this important investigation.The aim is to understand the incidence of CFS and to develop rational interventions to provide a scientific basis for improving the health status of college students and for governments to formulate health care measures.

The results of your responses are crucial to this investigation.The questionnaire consists of seven sections, each of which will be filled out by yourself. Be careful not to leave anything out.If you cannot answer a question for sure, please choose the answer that is closest to your true feeling.Don't hesitate to consult the investigator if you don't understand or have questions. It is estimated that the investigation will take you about 10 minutes.

9. Your personal data will be kept strictly confidential.The coded form of data entry will not expose your name, let alone spread your personal information.If we find any health problems after the investigation, we hope to provide you with further health care services.

Thank you very much for your participation in our research work!

Part 1: Basic information

Step 1: Name

2. Contact information (phone number)

Step 3: Gender

A. Male

B. Female

4. Age (years)

A. Under 22

B.22-25

C.26-29

D.30-33

E.34-37

F.37 above

5. Grade

A. Freshman year

B. Sophomore year

C. Junior year

D. Senior year

E. Graduate One

F. Study two

G. Study three

H. Boichi

I. Bo II

J. Botwo

6. Family location and region (setting options, such as: Hubei, Hunan, etc., 32 provinces in China, Hong Kong, Macao and Taiwan are not involved)

7. Major

A. Medical science

B. Arts

C. Management

D. Language

E. Science

F. Engineering

G. Economics

H. Literature and History

I. Others

8. Marital status

A Married B unmarried

有道文档翻译 pdf.youdao.com

9. Your ethnicity

① Han ② Tujia ③ Miao ④ Hui ⑤ Dong ⑥ Zhuang ⑦ others

10. Who do you live with at present?

① Roommates ② family ③ relatives ④ friends ⑤ live alone

11. Do you suffer from any of the following diseases?(Multiple choice)

A. Digestive diseases (e.g. Gastroesophageal reflux, peptic ulcer, pancreatitis, gallstones, ulcerative colitis, gastrointestinal bleeding, chronic gastroenteritis, irritable bowel syndrome, hemorrhoids, non-alcoholic fatty liver disease, etc.)

B.Cardiovascular and cerebrovascular diseases (such as coronary heart disease, valvular heart disease, transient ischemic attack, cerebral infarction, cerebral hemorrhage history)

C.Respiratory diseases (e.g., chronic bronchitis, chronic obstructive pulmonary disease, asthma, chronic rhinitis, chronic pharyngitis, chronic obstructive emphysema)

D.Reproductive system/specific diseases, skip to question 12 if male, or 13 if female

(1) Yes;(2) No

12. Male reproductive system/specific diseases:

Chronic prostatitis, chronic epididymitis, erectile dysfunction, adult varicocele, prostatic hyperplasia, infertility

13. Female reproductive system/specific diseases:

Chronic pelvic inflammatory disease, polycystic disease, dysmenorrhea, irregular menstruation, endometriosis, cervical cancer, breast hyperplasia/nodules, uterine fibroids, infertility

14. Do you have a history of the following chronic diseases?(Multiple choices)

A. Diabetes B. Hypertension C. hyperlipidemia D. Obesity E. hyperuricemia F. None of the above conditions

15. Are you suffering from any of the following diseases?(Multiple choice)

A. Diabetes B. hypertension C. hyperlipidemia D. obesity E. hyperuricemia F. None of the above

Part 2 Behavior and Lifestyle

1. How many times a week do you exercise on average

(1) more than 5 times per week (2) 3-5 times per week (3) 1-3 times per week (4) 1 time per week (5) never exercise

有道文档翻译 pdf.youdao.com

2. What kind of exercise do you usually do?(If you never exercise, skip this question)

(1) Running (2) swimming (3) Ball games (4) Yoga (5) Gym (6) Cycling (7) Others

3. How long do you exercise on average

(1) more than an hour (2)30 minutes to an hour (3) less than 30 minutes

4. Do you smoke (smoking is defined as smoking at least one cigarette a day for a cumulative period of more than six months)

A smoking B non-smoking C vaping

Your smoking status

(1) More than 1 pack per day (2) 1-1/2 pack per day (3) Less than 10 cigarettes per day

How many days per week are you exposed to secondhand smoke?(Secondhand smoke refers to the smoke exhaled by the smoker and emitted from the end of the cigarette when smoking)

(1) Daily (2) An average of 4-6 days per week (3) an average of 1-3 days per week (4) no

5. What kind of recreational activities do you mainly do after study and work?

(1) Indoor activities (computer, mobile phone games, watching TV, etc.) (2) outdoor activities (traveling, picnics, playing ball games, etc.)

6. Do you drink tea?

(1) Every day (2) often (3) occasionally (4) never

7. Do you drink alcohol?

(1) Every day (2) often (3) occasionally (4) never

8. The average amount of time you spend studying every day

(1) less than 1 hour (2)1-2 hours (3) 2-4 hours (4) more than 4 hours

9. How is your study stress?

(1) high (2) average (3) small (4) none

10. How many times do you usually have meals a day?

(1) once (2) twice (3) three times (4) more than four times

11. Do you have regular meal times

(1) You eat around the same time every day, (2) you occasionally eat at different times than usual, and (3) You eat when you're hungry

12. Do you ever overeat?

(1) Yes, often (2) Yes, occasionally (3) no

有道文档翻译 pdf.youdao.com

13. Do you limit your intake of sugar or sugary foods?(e.g. candy, cookies, preserves, sugary drinks, etc.)

A never B sometimes C often D always

14. You will maintain an ideal weight and avoid being too heavy or too light.

A always B sometimes C never

15. Do you stay up all night?

(1) No or occasionally (2) sometimes (3) often

16. Approximately how long each nap lasts

A Less than 30 minutes B less than an hour C more than an hour D no naps

17.Ways to turn to when you are in trouble:

(1) Relying only on yourself and not accepting help from others (2) rarely asking for help (3) sometimes asking for help from others (4) often asking for help from family, relatives and friends, and organizations when you are in trouble

18.For groups (such as caucuses, religious organizations, unions, student unions, etc.) to organize events, you would:

(1) Never participate (2) occasionally participate (3) often participate (4) Take the initiative to participate and be active

19.You find yourself easily relaxed and comfortable expressing emotions

A always B sometimes C never

20. Have you ever appeared in your daily life:

- Headache

A. No B. Very light C. moderate D. heavy E. severe

- Dizziness or fainting

A. No B. Very light C. moderate D. heavy E. severe

- Nausea or an upset stomach

A. No B. Very light C. moderate D. heavy E. severe

- Difficulty breathing

A. No B. Very light C. moderate D. heavy E. severe

- Tingling or tingling in the body

A. No B. Very light C. moderate D. heavy E. severe

有道文档翻译 pdf.youdao.com

- Feel heavy in your hands or feet

A. No B. Very light C. medium D. heavy E. severe

Part 3 FS-14

Physical Fatigue (Physical Fatigue)

1. Do you suffer from physical fatigue?

A Yes

B No

2. Do you need more rest?

A Yes

B No

3. Do you feel sleepy or drowsy?

A Yes

B No

4. Do you struggle when you start to do something?

A Yes

B No

5. Do you feel out of your depth when you start something?

A Yes

B No

6. Do you feel low on energy?

A Yes

B No

7. Do you feel that your muscle strength is less than before?

A Yes

B No

8. Are you feeling weak?

A Yes

B No

B) Mental Fatigue

有道文档翻译 pdf.youdao.com

9. Do you have trouble concentrating?

A Yes

B No

10. Are you as clear and sharp in your thinking as usual?

A Yes

B No

11. Do you use verbal dexterity when speaking?

A Yes

B No

12. Do you find it difficult to find the right words when speaking?

A is

B No

13. Is your memory as usual?

A is

B No

14. Do you still like to do the things you used to do?

A Yes

B No

CDC standard:

After 1.6 months, there is no obvious cause for recurrent burnout. The patient's current professional ability, educational ability, personal life ability and social activity ability are much lower than before the disease. At the same time, rest cannot effectively reduce the feeling of fatigue.

A is

B No

2. 4 or more of the following symptoms occur at the same time, and these symptoms last for more than 6 months:

(1) a noticeable and persistent feeling of fatigue;

有道文档翻译 pdf.youdao.com

(2) accompanied by anxiety and depression;

(3) poor sleep quality;

(4) reduced memory and cognitive function;

(5) poor concentration;

(6) swollen throat;

(7) pain and discomfort;

(8) neck stiffness;

(9) Swelling of axillary lymph nodes;

(10) multiple joint pain without obvious redness and swelling, muscle pain and discomfort

A Yes

B No

Part 4 Adjustment Scale for College Students

Some sentences about your personal situation are listed below.Please read each sentence carefully and choose the right one according to your own situation in the recent period of time.

1. There are always things I am interested in in everyday life.

A Disagree

B doesn't quite agree

C Not sure

D More agree

E Agree

2 If I had to choose again, I would still live my life as I do now.

A Disagrees

B doesn't quite agree

C Not sure

D More agree

E Agree

3 I always feel in a good mood.

有道文档翻译 pdf.youdao.com

A Disagrees

B doesn't quite agree

C Not sure

D More agree

E Agree

4. I seldom know what society needs for talent.

A Disagrees

B doesn't quite agree

C Not sure

D More agree

E Agree

5. I am often at a loss for things that frustrate me.

A Don't agree

B doesn't quite agree

C Not sure

D More agree

E Agree

6. I am satisfied with my postgraduate life.

A Disagree

B doesn't quite agree

C Not sure

D More agree

E Agree

7. I think my strengths outweigh my weaknesses.

A Disagree

B doesn't quite agree

C Not sure

D More agree

E Agree

8. A lot of people ask me to play with them.

有道文档翻译
pdf.youdao.com

A Don't agree

B doesn't quite agree

C Not sure

D More agree

E Agree

9. I can always find someone to keep me company when I don't want to do things alone.

A Disagrees

B doesn't quite agree

C Not sure

D More agree

E Agree

10. I know what's right for me.

A Disagree

B doesn't quite agree

C Not sure

D More agree

E Agree

11. I never feel lonely.

A Disagrees

B doesn't quite agree

C Not sure

D More agree

E Agree

12. I always look for my strengths and encourage myself by doing so.

A Don't agree

B doesn't quite agree

C Not sure

D More agree

E Agree

13. I don't know how to give compliments.

有道文档翻译 pdf.youdao.com

A Disagrees.

B doesn't quite agree

C Not sure

D More agree

E Agree

14. I don't make plans to achieve my career goals.

A Disagree

B doesn't quite agree

C Not sure

D More agree

E Agree

15. I don't know what to do when I have a conflict with others.

A: No.

B doesn't quite agree

C Not sure

D More agree

E Agree

16. I always compare my weaknesses with others' strengths.

A Don't agree

B doesn't quite agree

C Not sure

D More agree

E Agree

17. When I get hit, I think of the good in myself.

A Disagrees

B doesn't quite agree

C Not sure

D More agree

E Agree

18. I'm always full of energy and spirit.

有道文档翻译 pdf.youdao.com

A Disagrees

B doesn't quite agree

C Not sure

D More agree

E Agree

19. I think I am more capable than others.

A Disagree

B doesn't quite agree

C Not sure

D More agree

E Agree

20. Compared with my peers, I feel contented.

A Disagrees.

B doesn't quite agree

C Not sure

D More agree

E Agree

21. I'm not bothered about my appearance.

A Don't agree

B doesn't quite agree

C Not sure

D More agree

E Agree

There are many people willing to help me when I am in trouble.

A Don't agree

B doesn't quite agree

C Not sure

D More agree

E Agree

23. I know how to care about people.

有道文档翻译 pdf.youdao.com

A Disagrees.

B doesn't quite agree

C Not sure

D More agree

E Agree

24. I often adjust my emotional state by diverting my attention.

A Disagree

B doesn't quite agree

C Not sure

D More agree

E Agree

25. I will take various factors into consideration to determine my career goal.

A Disagree

B doesn't quite agree

C Not sure

D More agree

E Agree

26. I don't know how to let myself accept myself.

A Don't agree

B doesn't quite agree

C Not sure

D More agree

E Agree

27. I don't know what to do to cheer myself up.

A Don't agree

B doesn't quite agree

C Not sure

D) I agree with you.

E Agree

28. I think there are many unsatisfactory aspects in postgraduate life.

有道文档翻译 pdf.youdao.com

A Don't agree.

B doesn't quite agree

C Not sure

D More agree

E Agree

29. I am good at communicating with others verbally.

A Disagree

B doesn't quite agree

C Not sure

D More agree

E Agree

30. I often consciously prepare for my future job by taking part in social practice activities.

A Don't agree

B doesn't quite agree

C Not sure

D More agree

E Agree

31. When I am unhappy, I only complain.

A Disagree

B doesn't quite agree

C Not sure

D More agree

E Agree

32. I feel comfortable in my current dormitory relationship.

A Disagree

B doesn't quite agree

C Not sure

D More agree

E Agree

33. I don't know how to talk to strangers when I meet them.

有道文档翻译 pdf.youdao.com

A Don't agree

B doesn't quite agree

C Not sure

D More agree

E Agree

34. I can always find someone to talk to when I want to

A Disagrees.

B doesn't quite agree

C Not sure

D More agree

E Agree

35. I go out for a walk when I'm in a bad mood.

A Don't agree

B doesn't quite agree

C Not sure

D More agree

E Agree

36. You are in regular contact with your family

A Disagree

B doesn't quite agree

C Not sure

D More agree

E Agree

37. You feel good when family members call to check on you

A Disagree

B doesn't quite agree

C Not sure

D More agree

E Agree

38. You think graduate students should be thrifty

有道文档翻译
pdf.youdao.com

A Don't agree.

B doesn't quite agree

C Not sure

D More agree

E Agree

39. You think you are well off financially

A Don't agree

B doesn't quite agree

C Not sure

D More agree

E Agree

40. You believe that the other person arouses negative emotions in you during the course of the relationship

A Don't agree

B doesn't quite agree

C Not sure

D More agree

E Agree

Part 5 Depression Self-Rating Scale (SDS)

This is a depression questionnaire. You are asked to read each question carefully, make sense of it, and choose one of the answers that best fits your situation in the last week.

1. I feel down in the dumps.

○ Little or no time

○ Little time

○ Quite a bit of time

○ Most or all of the time

2. I think morning is the best part of the day.

有道文档翻译
pdf.youdao.com

○ No or very little time

○ Little time

○ Quite a bit of time

○ Most or all of the time

3. I burst into tears or feel like crying.

○ Little or no time

○ Little time

○ Quite a bit of time

○ Most or all of the time

4. I don't sleep well at night.

○ Little or no time

○ Little time

○ Quite a bit of time

○ Most or all of the time

5. I eat as much as I usually do.

○ Little or no time

○ Little time

○ Quite a bit of time

○ Most or all of the time

6. I am as happy as ever in close contact with the opposite sex.

○ Little or no time

○ Little time

○ Quite a bit of time

○ Most or all of the time

7. I noticed that I was losing weight.

○ Little or no time

○ Little time

○ Quite a bit of time

○ Most or all of the time

有道文档翻译 pdf.youdao.com

8. I suffer from constipation.

○ Little or no time

○ Little time

○ Quite a bit of time

○ Most or all of the time

9. My heart beats faster than usual.

○ Little or no time

○ Little time

○ Quite a bit of time

○ Most or all of the time

10. I feel tired for no apparent reason.

○ Little or no time

○ Little time

○ Quite a bit of time

○ Most or all of the time

11. My head is as clear as usual.

○ Little or no time

○ Little time

○ Quite a bit of time

○ Most or all of the time

12. I don't find it difficult to do the things I usually do.

○ Little or no time

○ Little time

○ Quite a bit of time

○ Most or all of the time

13. I feel restless and restless.

○ Little or no time

○ Little time

○ Quite a bit of time

有道文档翻译 pdf.youdao.com

○ Most or all of the time

14. I have hope for the future.

○ Little or no time

○ Little time

○ Quite a bit of time

○ Most or all of the time

15. I get angry and agitated more easily than usual.

○ Little or no time

○ Little time

○ Quite a bit of time

○ Most or all of the time

16. I find it easy to make the decision.

○ Little or no time

○ Little time

○ Quite a bit of time

○ Most or all of the time

17. I feel that I am a useful person who is needed.

○ Little or no time

○ Little time

○ Quite a bit of time

○ Most or all of the time

18. I've led an interesting life.

○ Little or no time

○ Little time

○ Quite a bit of time

○ Most or all of the time

19. I think others will be better off if I die.

○ Little or no time

○ Little time

有道文档翻译 pdf.youdao.com

○ Quite a bit of time

○ Most or all of the time

20. I'm still interested in the things that normally interest me.

○ Little or no time

○ Little time

○ Quite a bit of time

○ Most or all of the time

Part 6 Self-Rating Anxiety Scale (SAS)

This is a questionnaire of anxiety situations. Please read each question carefully, make clear the meaning, and choose the most suitable option among the answers according to your actual situation in the last week.

1. I feel more nervous and anxious than usual.

○ Little or no time

○ Little Time

○ Considerable time

○ Most or all of the time

2. I get scared for no reason.

○ Little or no time

○ Little Time

○ Considerable time

○ Most or all of the time

3. I tend to be upset or frightened.

○ Little or no time

○ Little Time

○ Considerable time

○ Most or all of the time

4. I think I'm going to go crazy.

○ Little or no time

有道文档翻译 pdf.youdao.com

○ Little Time

○ Considerable time

○ Most or all of the time

5. I think everything is fine and nothing bad will happen.

○ Little or no time

○ Little Time

○ Considerable time

○ Most or all of the time

6. My hands and feet are trembling.

○ Little or no time

○ Little Time

○ Considerable time

○ Most or all of the time

7. I suffer from headaches, head and neck pain, and back pain.

○ Little or no time

○ Little Time

○ Considerable time

○ Most or all of the time

8. I feel weak and tired easily.

○ Little or no time

○ Little Time

○ Considerable time

○ Most or all of the time

9. I feel calm and easy to sit still.

○ Little or no time

○ Little Time

○ Considerable time

○ Most or all of the time

10. I feel my heart beating very fast

有道文档翻译 pdf.youdao.com

○ Little or no time

○ Little Time

○ Considerable time

○ Most or all of the time

11. I suffer from bouts of dizziness.

○ Little or no time

○ Little Time

○ Considerable time

○ Most or all of the time

12. I have a faint attack or feel like I'm going to faint.

○ Little or no time

○ Little Time

○ Considerable time

○ Most or all of the time

13. I can breathe in and out easily.

○ Little or no time

○ Little Time

○ Considerable time

○ Most or all of the time

14. My hands and feet are numb and tingling.

○ No or very little time

○ Little Time

○ Considerable time

○ Most or all of the time

15. I suffer from stomachaches and indigestion.

○ Little or no time

○ Little Time

○ Considerable time

○ Most or all of the time

有道文档翻译 pdf.youdao.com

16. I urinate frequently.

○ Little or no time

○ Little Time

○ Considerable time

○ Most or all of the time

17. My hands and feet are always dry and warm.

○ Little or no time

○ Little Time

○ Considerable time

○ Most or all of the time

18. I often blush and get hot.

○ Little or no time

○ Little Time

○ Considerable time

○ Most or all of the time

19. I fall asleep easily and sleep well all night

○ Little or no time

○ Little Time

○ Considerable time

○ Most or all of the time

20. I often have nightmares.

○ Little or no time

○ Little Time

○ Considerable time

○ Most or all of the time

有道文档翻译 pdf.youdao.com

Part 7 Pittsburgh Sleep Quality refers to Quantity (PSQI) table

Some sentences about how you sleep are listed below.Please read each sentence carefully and choose the right one based on your sleep conditions in the recent period.

1 Nearly 1 month, go to bed at night usually in

A. before 21.00 B. 21.00-22.00 C. 22.00-24:00 C. After midnight C.

2 For nearly 1 month, it usually takes min from going to bed to falling asleep

A.≤ 15minB.16~30min C.31~60min D.≥ 60min

3 For nearly 1 month, getting up is usually in the morning

A. before 6am B. 6-8am C. 8-10am D. after 10am

4 For nearly 1 month, actual sleep per night is usually (not equal to bed time)

A. Less than 6h B.6h-8h C.8h-10h D. more than 10h

5 For nearly 1 month, the following conditions affect sleep and worry

a. Difficulty falling asleep (unable to fall asleep within 30 minutes)

A. None B.< once/week C. 1~2 times/week D.≥3 times/week

b. Easy to wake up or wake up early at night

A. None B.< once/week C. 1~2 times/week D.≥3 times/week

c. Go to the bathroom at night

A. None B.< once/week C. 1~2 times/week D.≥3 times/week

d. Dyspnea

A. None B.< once/week C. 1~2 times/week D.≥3 times/week

e. Cough or snore loudly

A. None B.< once/week C. 1~2 times/week D.≥3 times/week

f. Feeling cold

A. None B.< once/week C. 1~2 times/week D.≥3 times/week

g. Feeling hot

A. None B.< once/week C. 1~2 times/week D.≥3 times/week

h. Have nightmares

A. None B.< once/week C. 1~2 times/week D.≥3 times/week

i. Pain and discomfort

A. None B.< once/week C. 1~2 times/week D.≥3 times/week

j. Other things that affect your sleep (if any, please explain:)

A. None B.< once/week C. 1~2 times/week D.≥3 times/week

6. For nearly 1 month, overall, do you think your sleep quality:

pdf.youdao.com

A. Very good B. good C. poor D. very poor

7. In the past 1 month, your use of drug hypnosis:

A. None B.< once/week C. 1~2 times/week D.≥3 times/week

8. In the past 1 month, have you often felt sleepy?

A. None B.< once/week C. 1~2 times/week D. ⩾3 times/week

9. Do you have insufficient energy to do things in the past month?

A. No B. Occasionally C. sometimes D. often

有道文档翻译
pdf.youdao.com
