# Supplementary material for: A description of the current status of chronic fatigue syndrome and associated factors among university students in Wuhan, China
Source: Front Psychiatry. 2023 Jan 12;13:1047014. doi: 10.3389/fpsyt.2022.1047014 (PMC9877457; doi:10.3389/fpsyt.2022.1047014)
Supplement: Supplementary file 4 [file Table_1.DOCX]

**supplementary table 1 Comparison of FS-14 scores of college students with the general situation**

| **Variable** | **n** | **Physical Fatigue** | **Mental Fatigue** | **Total** |
| --- | --- | --- | --- | --- |
| **gender** |  |  |  |  |
| male | 922 | 3.4±2.7 | 1.7±1.4 | 5.0±3.6 |
| female | 800 | 3.4±2.5 | 1.4±1.2 | 4.8±3.3 |
| *Z* |  | 0.62 | -4.23 | -1.06 |
| *P* |  | 0.53 | ＜0.05 | 0.29 |
| **age** |  |  |  |  |
| ＜19 | 326 | 3.3±2.5 | 1.6±1.3 | 4.9±3.4 |
| 19-25 | 1362 | 3.4±2.6 | 1.5±1.3 | 4.9±3.4 |
| ＞25 | 104 | 3.6±2.7 | 1.8±1.5 | 5..4±3.5 |
| *X2* |  | 1.33 | 4.90 | 1.97 |
| *P* |  | 0.51 | 0.08 | 0.37 |
| **marital status** |  |  |  |  |
| married | 81 | 3.8±2.6 | 1.5±1.4 | 5.3±3.3 |
| spinsterhood | 1711 | 3.4±2.6 | 1.5±1.3 | 4.9±3.4 |
| *Z* |  | 1.45 | -0.59 | 1.14 |
| *P* |  | 0.15 | 0.56 | 0.26 |
| **physical training** |  |  |  |  |
| more than 5 times a week | 179 | 2.8±2.6 | 1.4±1.2 | 4.2±3.3 |
| 3-5 times a week | 299 | 3.1±2.5 | 1.4±1.3 | 4.6±3.4 |
| 1-3 times a week | 780 | 3.3±2.5 | 1.4±1.3 | 4.7±3.3 |
| 1 times per week | 383 | 4.0±2.6 | 1.8±1.4 | 5.8±3.5 |
| Never exercise | 151 | 4.0±2.7 | 1.7±1.4 | 5.7±3.5 |
| *X2* |  | 42.79 | 22.64 | 22.63 |
| *P* |  | ＜0.0001 | ＜0.0001 | ＜0.0001 |
| **smoke** |  |  |  |  |
| yes | 64 | 3.5±2.5 | 1.58±1.34 | 5.1±3.3 |
| no | 1728 | 3.4±2.6 | 1.53±1.31 | 4.9±3.4 |
| *Z* |  | 0.29 | 0.31 | 0.44 |
| *P* |  | 0.77 | 0.76 | 0.66 |
| **drink tea** |  |  |  |  |
| yes | 1260 | 3.4±2.6 | 1.5±1.3 | 4.9±3.4 |
| no | 532 | 3.5±2.6 | 1.6±1.3 | 5.0±3.5 |
| *Z* |  | 0.39 | 4.05 | 0.95 |
| *P* |  | 0.69 | ＜0.05 | 0.33 |
| **drink** |  |  |  |  |
| yes | 641 | 3.6±2.6 | 1.6±1.3 | 5.2±3.5 |
| no | 1151 | 3.3±2.6 | 1.5±1.3 | 4.8±3.4 |
| *Z* |  | 6.07 | 1.97 | 6.78 |
| *P* |  | ＜0.05 | ＜0.05 | ＜0.05 |
| **study** |  |  |  |  |
| Less than 1 hour | 190 | 3.9±2.7 | 1.9±1.5 | 5.8±3.6 |
| 1 to 2 hours | 503 | 3.7±2.6 | 1.6±1.3 | 5.3±3.5 |
| 2-4 hours | 497 | 2.9±2.4 | 1.5±1.2 | 4.4±3.2 |
| More than 4 hours | 602 | 3.4±2.6 | 1.4±1.3 | 4.9±3.5 |
| *X2* |  | 27.05 | 17.47 | 26.04 |
| *P* |  | ＜0.0001 | ＜0.05 | ＜0.0001 |
| **Dining** |  |  |  |  |
| Meal times are similar every day | 1025 | 3.1±2.5 | 1.4±1.3 | 4.5±3.3 |
| Occasionally not at the usual meal time | 591 | 3.8±2.6 | 1.6±1.3 | 5.4±3.4 |
| no specific meal time to eat | 176 | 4.1±2.7 | 2.0±1.5 | 6.0±3.7 |
| *X2* |  | 35.71 | 31.41 | 43.15 |
| *P* |  | ＜0.0001 | ＜0.0001 | ＜0.0001 |
| **Overeating** |  |  |  |  |
| yes | 712 | 4.1±2.7 | 1.8±1.4 | 6.0±3.5 |
| no | 1080 | 2.9±2.5 | 1.3±1.2 | 4.3±3.2 |
| *Z* |  | 9.42 | 7.71 | 10.01 |
| *P* |  | ＜0.0001 | ＜0.0001 | ＜0.0001 |
| **snacks** |  |  |  |  |
| yes | 1275 | 3.6±2.6 | 1.6±1.3 | 5.1±3.4 |
| no | 517 | 3.0±2.6 | 1.4±1.3 | 4.4±3.4 |
| *Z* |  | 4.22 | 2.8 | 4.21 |
| *P* |  | ＜0.0001 | ＜0.05 | ＜0.0001 |
| **dietary structure** |  |  |  |  |
| A menear diet | 290 | 4.2±2.7 | 1.9±1.4 | 6.1±3.6 |
| A vegetarian diet | 104 | 3.5±2.4 | 1.8±1.3 | 5.2±3.3 |
| Mix meat quality | 1398 | 3.2±2.6 | 1.4±1.3 | 4.7±3.4 |
| *X2* |  | 33.55 | 30.25 | 40.3 |
| *P* |  | ＜0.0001 | ＜0.0001 | ＜0.0001 |
| **milky tea** |  |  |  |  |
| frequently | 169 | 4.2±2.6 | 1.6±1.3 | 5.9±3.4 |
| occasionally | 1329 | 3.4±2.5 | 1.5±1.3 | 4.9±3.4 |
| never | 294 | 3.1±2.7 | 1.4±1.3 | 4.6±3.5 |
| *X2* |  | 21.33 | 3.40 | 17.34 |
| *P* |  | ＜0.0001 | 0.18 | ＜0.05 |
| **pickles** |  |  |  |  |
| frequently | 113 | 4.4±2.8 | 1.9±1.4 | 6.3±3.8 |
| occasionally | 1364 | 3.4±2.6 | 1.5±1.3 | 4.9±3.4 |
| never | 315 | 3.2±2.6 | 1.4±1.3 | 4.6±3.4 |
| *X2* |  | 16.78 | 11.61 | 17.96 |
| *P* |  | ＜0.05 | ＜0.05 | ＜0.0001 |
| **Pay attention to the nutrition facts list** |  |  |  |  |
| frequently | 404 | 3.3±2.6 | 1.5±1.3 | 4.8±3.5 |
| occasionally | 898 | 3.3±2.6 | 1.5±1.3 | 4.8±3.4 |
| never | 490 | 3.7±2.6 | 1.6±1.4 | 5.3±3.5 |
| *X2* |  | 6.71 | 2.88 | 7.35 |
| *P* |  | ＜0.05 | 0.24 | ＜0.05 |
| **Accused of sugar** |  |  |  |  |
| never | 524 | 3.8±2.6 | 1.7±1.3 | 5.5±3.5 |
| occasionally | 826 | 3.3±2.6 | 1.4±1.3 | 4.8±3.3 |
| frequently | 442 | 3.2±2.6 | 1.5±1.3 | 4.7±3.4 |
| *X2* |  | 13.16 | 14.00 | 16.53 |
| *P* |  | ＜0.05 | ＜0.05 | ＜0.05 |
| **maintain the weight** |  |  |  |  |
| frequently | 532 | 3.2±2.5 | 1.4±1.3 | 4.6±3.3 |
| occasionally | 799 | 3.5±2.7 | 1.5±1.3 | 5.0±3.5 |
| never | 461 | 3.6±2.6 | 1.7±1.4 | 5.2±3.5 |
| *X2* |  | 5.69 | 6.43 | 7.48 |
| *P* |  | 0.06 | ＜0.05 | ＜0.05 |
| **Overnight** |  |  |  |  |
| No or occasionally | 1275 | 3.2±2.5 | 1.4±1.3 | 4.6±3.3 |
| sometimes | 427 | 3.8±2.8 | 1.8±1.3 | 5.5±3.6 |
| frequently | 90 | 4.3±2.7 | 2.2±1.5 | 6.4±3.7 |
| *X2* |  | 19.98 | 44.24 | 32.58 |
| *P* |  | ＜0.0001 | ＜0.0001 | ＜0.0001 |
| **siesta** |  |  |  |  |
| yes | 1604 | 3.4±2.6 | 1.5±1.3 | 4.9±3.4 |
| no | 188 | 3.5±2.8 | 1.7±1.3 | 5.2±3.5 |
| *Z* |  | 0.57 | 1.63 | 1.09 |
| *P* |  | 0.28 | 0.05 | 0.14 |
| **Whether to ask for help when encountering trouble** |  |  |  |  |
| never | 170 | 4.2±2.8 | 1.9±1.4 | 6.2±3.7 |
| rarely | 462 | 3.4±2.6 | 1.7±1.4 | 5.1±3.6 |
| sometimes | 740 | 3.4±2.5 | 1.5±1.3 | 4.9±3.2 |
| frequently | 420 | 3.2±2.6 | 1.2±1.2 | 4.4±3.4 |
| *X2* |  | 20.84 | 39.44 | 31.24 |
| *P* |  | ＜0.0001 | ＜0.0001 | ＜0.0001 |
| **Whether to participate in group activities** |  |  |  |  |
| never | 205 | 3.8±2.8 | 1.8±1.5 | 5.7±3.8 |
| occasionally | 1091 | 3.4±2.6 | 1.6±1.3 | 5.0±3.4 |
| frequently | 496 | 3.3±2.5 | 1.4±1.2 | 4.6±3.3 |
| *X2* |  | 5.71 | 15.96 | 9.48 |
| *P* |  | 0.06 | ＜0.05 | ＜0.05 |
| **Feel comfortable expressing your feelings** |  |  |  |  |
| all the time | 438 | 2.9±2.5 | 1.2±1.2 | 4.1±3.3 |
| sometimes | 1188 | 3.5±2.6 | 1.6±1.3 | 5.0±3.4 |
| never | 166 | 4.3±2.7 | 2.2±1.4 | 6.5±3.6 |
| *X2* |  | 39.8 | 74.58 | 62.77 |
| *P* |  | ＜0.0001 | ＜0.0001 | ＜0.0001 |
| **Whether there is a headache in daily life** |  |  |  |  |
| no | 727 | 2.4±2.4 | 1.2±1.2 | 3.7±3.1 |
| yes | 1065 | 4.1±2.5 | 1.7±1.4 | 5.8±3.4 |
| *Z* |  | -13.59 | -7.86 | 13.43 |
| *P* |  | ＜0.0001 | ＜0.0001 | ＜0.0001 |
| **dizziness** |  |  |  |  |
| no | 1289 | 3.0±2.5 | 1.4±1.3 | 4.4±3.3 |
| yes | 503 | 4.5±2.6 | 1.9±1.3 | 6.4±3.4 |
| *Z* |  | 10.64 | 8.06 | 11.04 |
| *P* |  | ＜0.0001 | ＜0.0001 | ＜0.0001 |
| **nausea** |  |  |  |  |
| no | 870 | 2.5±3.4 | 1.3±1.2 | 3.7±3.1 |
| yes | 922 | 4.3±2.5 | 1.8±1.3 | 6.1±3.3 |
| *Z* |  | -15.38 | -8.79 | -15.04 |
| *P* |  | ＜0.0001 | ＜0.0001 | ＜0.0001 |
| **dyspnea** |  |  |  |  |
| no | 1389 | 3.0±2.5 | 1.3±1.2 | 4.4±3.2 |
| yes | 403 | 4.8±2.6 | 2.1±1.4 | 6.9±3.4 |
| *Z* |  | 11.65 | 10.7 | 12.74 |
| *P* |  | ＜0.0001 | ＜0.0001 | ＜0.0001 |
| **Numbness or tingling in the body** |  |  |  |  |
| no | 1337 | 3.0±2.5 | 1.3±1.2 | 4.3±3.2 |
| yes | 455 | 4.7±2.5 | 2.1±1.4 | 6.7±3.4 |
| *Z* |  | 11.69 | 10.12 | 12.52 |
| *P* |  | ＜0.0001 | ＜0.0001 | ＜0.0001 |
| **Hands and feet sink** |  |  |  |  |
| no | 1275 | 2.8±2.4 | 1.3±1.2 | 4.1±3.1 |
| yes | 517 | 5.0±2.5 | 2.1±1.4 | 7.0±3.3 |
| *Z* |  | 15.63 | 10.6 | 15.78 |
| *P* |  | ＜0.0001 | ＜0.0001 | ＜0.0001 |

**supplementary table 2 Assignment and variables**

| **variable** | **assignment** |
| --- | --- |
| **physical training** | 0=More than 5 times a week |
|  | 1=3-5 times a week |
|  | 2=1-3 times a week |
|  | 3=1 times per week |
|  | 4=never |
| **drink** | 0=no |
|  | 1=yes |
| **study** | 0=Less than 1 hour |
|  | 1=1-2 hours |
|  | 2=2-4 hours |
|  | 3=More than 4 hours |
| **Overnight** | 0=No or occasionally |
|  | 1=sometimes |
|  | 2=frequently |
| **Overeating** | 0=no |
|  | 1=yes |
| **depression** | 0=no |
|  | 1=yes |
| **anxiety** | 0=no |
|  | 1=yes |
| **sleep disorders** | 0=no |
|  | 1=yes |
| **CFS** | 0=no |
|  | 1=yes |
